# Pilot study: The effect of eight weeks of aerobic training in the two phases of light and dark circadian rhythm on genes (OCT4, NANOG, SOX2, REX1) in bone tissue of diabetic mice

**DOI:** 10.1371/journal.pone.0353987

**Published:** 2026-07-27

**Authors:** Maryam Janbozorgi, Asma Taheri, Masoumeh Hosseinzadeh, Sahar Ghafaripur

**Affiliations:** 1 Assistant Professor, Department of Sports Physiology, Faculty of Sports Sciences, Shahid Chamran University of Ahvaz, Ahvaz, Iran; 2 Instructor, Department of Sport Physiology, Faculty of Sport Sciences, Shahid Chamran University of Ahvaz, Ahvaz, Iran; 3 Graduate of Exercise Physiology, Shahid Chamran University of Ahvaz, Ahvaz, Iran; National Research Centre, EGYPT

## Abstract

**Introduction:**

Diabetes mellitus impairs bone metabolism through chronic hyperglycemia, oxidative stress, and inflammation, leading to reduced osteoblast activity and downregulation of key pluripotency genes such as Octamer-binding transcription factor 4 (OCT4), Nanog homeobox (NANOG), SRY-box transcription factor 2 (SOX2), and Reduced expression protein 1 (REX1).The present study investigated whether the timing of aerobic exercise (morning vs. evening) influences the expression of pluripotency genes in the bone tissue of diabetic mice.

**Methods:**

Eighteen male NMRI mice were assigned to healthy control, diabetic control, or diabetic exercise groups. Diabetes was induced using a high-fat diet combined with low-dose streptozotocin. Aerobic training was performed for eight weeks at either ZT3 (morning) or ZT15 (evening), five sessions per week, at 50–60% Vmax. Blood glucose, insulin, HOMA-IR, and maximum running speed were assessed, and gene expression in bone tissue was measured using qRT-PCR. Data were analyzed using one-way ANOVA with Tukey post hoc testing (p < 0.05).

**Results:**

Diabetic mice exhibited significant increases in glucose, insulin, and HOMA-IR, along with marked reductions in pluripotency gene expression, particularly at ZT3. Aerobic exercise significantly improved metabolic parameters and partially restored gene expression, with the greatest enhancement observed at ZT15. SOX2 and REX1 showed the most robust recovery under evening training conditions.

**Conclusion:**

The findings indicate that circadian timing modulates the beneficial effects of aerobic exercise on pluripotency gene expression in diabetic bone tissue. Evening exercise (ZT15) exerts superior restorative effects, suggesting that chronobiology aligned training may serve as an effective strategy for improving bone regenerative potential in diabetes.

## Introduction

Type 1 and 2 diabetes mellitus, as one of the most common chronic metabolic diseases in the world, affect millions of people annually. This disease is associated with impaired blood sugar regulation, insulin resistance, and chronic systemic inflammation, which can cause multiple disorders in body tissues, including bone tissue [1]. Studies have shown that diabetes leads to reduced bone mineral density (BMD), impaired osteoblast cell activity, and increased risk of bone fracture [2]. Studies have shown that diabetes leads to reduced bone density and increased risk of fracture by reducing osteoblast activity, increasing osteoclast activity, and inhibiting the differentiation of mesenchymal stem cells (MSCs) into osteogenic cells [3]. At the molecular level, diabetes leads to reduced expression of key genes related to pluripotency and self-renewal of cells by causing oxidative stress, chronic inflammation, and disruption of cell signaling pathways. OCT4 (Octamer-binding transcription factor 4), NANOG (Nanog homeobox), SOX2 (SRY-box transcription factor 2), and REX1 (Reduced expression protein 1) genes are among the most important markers of pluripotent stem cells that play a role in maintaining the differentiation and regeneration ability of stem cells [4,5]. Reduced expression of these genes has been reported in diabetic conditions, and this could be one of the main reasons for poor bone regeneration in diabetic patients [6]. The expression of these genes in bone tissue can be an indicator of its repair and regeneration potential. Studies have shown that pathological conditions such as diabetes can be associated with reduced expression of these genes [7]; while exercise may increase their expression by stimulating cell signaling pathways and help tissue regeneration [8]. In the meantime, one of the effective non-pharmacological strategies for improving the physiological status of diabetic patients is regular exercise, especially aerobic exercise. Aerobic exercise can have positive effects on bone structure and function by improving insulin sensitivity, lowering blood glucose levels, and reducing inflammatory processes [6]. However, recent findings in exercise physiology and chronobiology (chronobiology) suggest that the time of day (morning or evening) of exercise may have different effects on cellular and genetic responses [9,10]. Exercise not only increases insulin sensitivity and lowers blood sugar, but also has important effects on the expression of genes related to tissue repair and cell differentiation [6]. Recent studies have shown that aerobic exercise can increase the expression of OCT4, NANOG, SOX2, and REX1 genes in various tissues, including muscle and bone marrow, and enhance the capacity for cell repair [11]. This increase in gene expression is likely to occur through the activation of signaling pathways such as PI3K/Akt, AMPK, and Wnt/β-catenin [11] In addition to the type and intensity of exercise, the time of day (morning or evening) of exercise can also play an important role in the body’s physiological and molecular responses. According to findings in the field of chronobiology, the human body has specific circadian rhythms that affect metabolism, hormones, and gene expression [8]. Evidence suggests that exercise training at different times of the day may lead to different responses in gene expression levels [9]. Given the importance of these issues, the present study was designed to investigate the effect of eight weeks of regular aerobic exercise at two different times of the day (morning and evening) on the expression of OCT4, NANOG, SOX2, and REX1 genes in the bone tissue of diabetic mice. This research attempts to provide a scientific answer to the question of whether exercise time can affect the expression of essential genes and bone remodeling in diabetic conditions.

## Methods

The present study is experimental in terms of laboratory implementation and simple random sampling. This study was designed as a pilot experimental animal study because of the limited sample size, with the primary aim of providing preliminary evidence regarding the effects of exercise timing on pluripotency-related gene expression in diabetic bone tissue. Eighteen adult male NMRI mice, eight to ten weeks old, with an average weight of 26 ± 3.22 grams, were purchased from the Laboratory Animal Breeding Center of Ahvaz Jundishapur University of Medical Sciences. Mice were selected because the streptozotocin-induced diabetic mouse model is well established for studying diabetes-related metabolic and molecular alterations, including gene expression. Additionally, mice offer broader molecular reagent compatibility for pluripotency-related gene analysis. In order to induce diabetes, a combination of high-fat diet and low-dose streptozotocin (STZ) was used. Based on this method, mice in the diabetic groups received HFD containing 60% of total calories from fat, 24% protein, and 26% carbohydrate, providing 5.2 kcal/g for 5 weeks. In contrast, control mice received a regular standard diet containing 5.1% fat, 23% protein, and 50.3% carbohydrate, with an energy density of 3.1 kcal/g. At the end of this period, they received STZ (20 mg per kg of body weight) [12] once by intraperitoneal injection. STZ was prepared daily in citrate buffer pH = 5 and injected over 30 minutes. Five days after STZ injection, mice whose fasting glucose was greater than 126 mg/dl were considered diabetic and randomly assigned to 6 groups total based on weight homogenization [13]. Healthy control groups were also purchased at the same time as the diabetic group and fed with a normal diet and received STZ solvent. The mice were kept in groups of three in transparent polycarbonate cages measuring 30 cm in length, width and height, manufactured by Razi Rad Company, in an environment with a temperature of 22 ± 2 °C, humidity of 45–55% and a 12:12 hour light-dark cycle. During the study, standard pellet food and water were freely available to the samples.

### Experimental groups

The animals were divided into six experimental groups as follows:

- Healthy control group at Zeitgeber Time 3 (ZT3)
- Healthy control group at Zeitgeber Time 15 (ZT15)
- Diabetic control group at Zeitgeber Time 3 (ZT3)
- Diabetic control group at Zeitgeber Time 15 (ZT15)
- Diabetic training group at Zeitgeber Time 3 (ZT3)
- Diabetic training group at Zeitgeber Time 15 (ZT15)

### Determination of maximum speed (Vmax)

At the end of the adaptation week, the fourth and eighth weeks of the main training, the maximum speed test was taken from all groups. In the training groups, the intensity of the training was adjusted by training in the measured range – using the Vmax obtained at the beginning and the fourth week of training. The mice of the different groups were placed on the treadmill and warmed up for 5 minutes at a speed of 6 m/min. Then the speed was increased every 2 minutes by 2 m/min. Until the mice were unable or unwilling to continue the task. The final running speed of the mice was recorded as their maximum speed and to prevent any disturbance and its effect on the performance of the mice and the results of the study, the maximum speed for each group was measured per hour corresponding to the same group [14,15]

### Endurance training (moderate-intensity continuous training)

One week after ensuring the induction of diabetes, the training groups trained at two different times of the day; To determine the time of day training, the light onset time was 6 am (ZT0) and the dark onset time was 6 pm (ZT12), and the training time was three hours after the light onset at 9 am (ZT3) and three hours after the dark onset at 9 pm (ZT15) [16]. Before the main training program, the mice were gradually familiarized with the treadmill for one week to adapt to the treadmill. The training program was adjusted and performed in the training groups with a moderate intensity of 50–60% Vmax for 80 minutes per session. This training was held for 8 weeks and 5 sessions per week [14]. Weight and blood glucose were assessed weekly. Sampling. 48 hours after the end of the eight-week training period, the mice were transferred to the sampling room and anesthetized with an intraperitoneal injection ketamine (30–50 mg/kg body weight) and xylazine (3–5 mg/kg body weight). Blood samples were obtained directly from the heart and centrifuged at 5000 rpm for 5 minutes to separate the serum samples. Bone tissue was also collected at the same time and transferred to a −70 freezer for further testing. Since several time points are required to detect changes in the phase or amplitude of the molecular clock, each group was analyzed at its respective training hour [17].

### Measurement of insulin resistance index

To calculate the insulin resistance index (HOMA-IR), blood glucose was assessed by glucose oxidase and spectrophotometry (Pars Azmoun, Iran, and insulin was assessed by species-specific ELISA (Bioassay Technology, China) with an interassay accuracy of 3.4% and an intraassay accuracy of 4.3%. The following formula was used to measure insulin resistance:

Homa-IR= (Fasting Insulin(μU/ml) *Fasting Glucose(mmol/L))/22.5

An increase or decrease in HOMA-IRin diabetic subjects compared to healthy mice was considered to indicate an increase or decrease in insulin resistance, respectively.

### Ethical approval and animal welfare

All animal procedures were approved by the Committee for Ethics in Animal Experiments at Shahid Chamran University (License No. EE/1401.2.24.173079/scu.ac.ir) and were conducted in accordance with the National Institutes of Health Guide for the Care and Use of Laboratory Animals (NIH Publication No. 86–23).

All efforts were made to minimize animal suffering throughout the study. Animals were housed under controlled environmental conditions (22 ± 2°C, 45–55% humidity, 12:12 h light/dark cycle) with free access to food and water. Mice were gradually acclimatized to treadmill exercise before the intervention, and their health status was monitored regularly throughout the experimental period. All terminal procedures were performed under deep ketamine/xylazine anesthesia, and animals were euthanized by exsanguination while remaining deeply anesthetized.

### Real-Time Quantitative RT-PCR

To evaluate changes in the expression level of OCT4, NANOG, SOX2 and REX1 genes, realtime PCR was performed using qPCRTM Green Master Kit for SYBR Green I (Jena Biosciense, Germany) on a Lightcycl Detection System (Roche, USA). Relative expression level of the OCT4, NANOG, SOX2 and REX1 transcripts were compared to mice GAPDH as housekeeping gene.

Reactions were performed in a 12.5 μL mixture containing 6.25 μL qPCRTM Green Master Kit for SYBR Green I (Jena Biosciense, Germany), 0.25 μL of each primer (200 nM), 3 μL cDNA (100 ng), and 2.25 μL nuclease-free water. The PCR protocol used consisted of a 5 min denaturation at 94°C followed by 45 cycles of 94°C for 15 s, 60°C for 30 s. Reactions were performed in triplicate. Two separate reactions without cDNA or with RNA were performed in paraμLel as controls. Relative quantification was performed according to the comparative 2^-ΔΔCt method and using Lightcycler 96 software. all qPCR analysis was performed according to The minimum information for publication of quantitative real-time PCR experiments (MIQE) guideline [18].

### Primer design

The sequences OCT4, NANOG, SOX2, REX1 and GAPDH genes were obtained from NCBI database and primer sets were designed via GeneRunner and Primer Express software v.3.0 (Applied Biosystems, Foster City, USA) and analyzed in Basic Local Alignment Search Tool to avoid homology with other genome region. Oligonucleotide sequences are shown in Table 1.

**Table 1:** Characteristics of primers which were used for Real-Time PCR analysis.

GENE NAME	SEQUENCE	accession NO
OCT4-mice-F	CGAACTAGCATTGAGAACCGTG	NM_001252452.1
OCT4-mice-R	CCATACTCGAACCACATCCTTCT	
GAPDH-mice-F	CATCACTGCCACCCAGAAGACTG	NM_008084
GAPDH-mice-R	ATGCCAGTGAGCTTCCCGTTCAG	
NANOG-mice-F	GAACGCCTCATCAATGCCTGCA	NM_028016
NANOG-mice-R	GAATCAGGGCTGCCTTGAAGAG	
SOX2-mice-F	AACGGCAGCTACAGCATGATGC	NM_011443
SOX2-mice-R	CGAGCTGGTCATGGAGTTGTAC	
REX1-mice-F	GAGACTGAGGAAGATGGCTTCC	NM_009556
REX1-mice-R	CTGGCGAGAAAGGTTTTGCTCC	

### Statistical analyses

Data analyses were done using the SPSS 26.0 software package (SPSS Inc., Chicago, IL, USA). One-way ANOVA was used to test differences between various means (post hoc analysis Tukey test). all experimental data were presented as the mean ± SEM. The level of significance for all tests was set at p\ 0.05.

## Results

The results show that the effect of aerobic exercise on the measured parameters of diabetic mice is significant at the one percent level. Comparison of the mean of the evaluated variables, after the intervention, in the different research groups is presented in Table 2.

**Table 2:** Mean and standard deviation of the studied variables after intervention.

	Weight	Homa-IR	Insulin	Glucose	Vmax
	G	μU/ml* mmol/l	mU/L	Mg/dl	m/min
**CH-ZT3**	33.00±3.60^b^	9.99±0.85 ^f^	40.08±3.61 ^e^	101.18±4.10 ^e^	15.16±1.04 ^b^
**CH-ZT15**	34.00±2.64 ^b^	11.83±1.74 ^e^	41.10±5.25 ^e^	116.40±4.22 ^d^	15.50±1.32 ^b^
**CD-ZT3**	41.33±1.52 ^a^	31.17±0.79 ^b^	88.04±5.36 ^b^	143.58±5.09 ^b^	14.83±1.60 ^b^
**CD-ZT15**	40.66±3.05^a^	37.80±3.04 ^a^	97.53±6.43 ^a^	157.33±5.45 ^a^	15.60±0.91 ^b^
**TD-ZT3**	34.33±1.52 ^b^	25.27±3.38 ^c^	71.32±7.12 ^c^	143.20±5.63 ^b^	24.66±1.15 ^a^
**TD-ZT15**	36.33±1.55 ^b^	20.10±2.60 ^d^	63.80±5.87 ^d^	127.40±3.36 ^c^	24.80 ± 1.70 ^a^

Non-identical Latin letters above the columns indicate that their differences are significant in the TUKEY post hoc test. CH-ZT3 = healthy control group light phase, CH-ZT15 = healthy control group dark phase, CD-ZT3 = diabetic control group light phase, CD-ZT15 = diabetic control group dark phase, TD-ZT3 = diabetic exercise group light phase, TD-ZT15 = diabetic exercise group dark phase. HOMA-IR: Index of insulin resistance.

The results of the post hoc test between the study groups showed a significant increase in the levels of glucose, insulin, HOMA-IR and weight in diabetic mice compared to healthy control mice (P < 0.05). The effect of aerobic training on the changes in the levels of these variables in the training groups compared to the diabetic groups was significant, and the decreasing trend in the levels of these variables in the trained mice was significant (P < 0.05). The changes in the levels of glucose, insulin and HOMA-IR variables in the two dark and light phases in all the study groups showed a significant difference (P < 0.05). However, the changes in weight in the two dark and light phases were not significant (P > 0.05). The evaluation of the changes in maximum speed between the study groups showed a significant effect of aerobic training on increasing the maximum speed in the trained mice compared to the other groups (P < 0.05). Changes in maximum speed in the two phases of darkness and light were not significant (P > 0.05).

**Fig 1:**
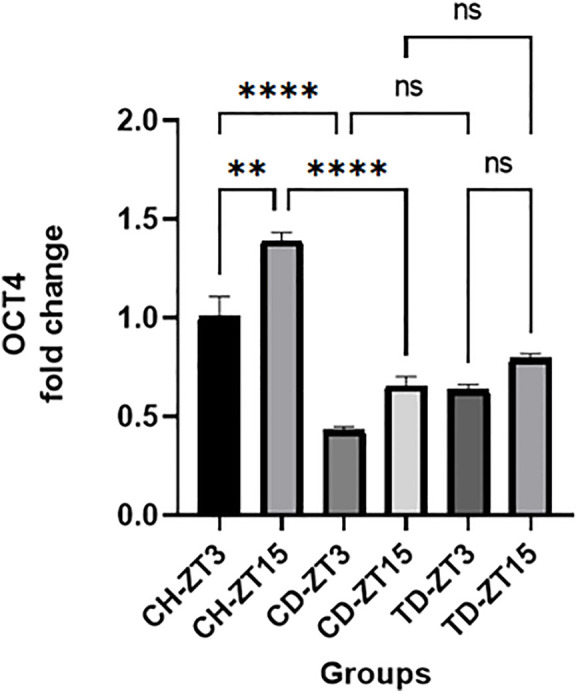
OCT4 gene level expressed in different groups. Data are mean ± SD. pairwise compairsons Between bars indicates rate of significant difference at p\0.05.

Fig 1 shows the relative expression levels of the OCT4 gene in the experimental groups.Quantitative analysis of OCT4 expression revealed distinct temporal and condition-dependent variations across the experimental groups. In the CH-ZT15 group, OCT4 levels demonstrated a pronounced elevation (mean ≈ 1.6 ± 0.12), and this increase reached statistical significance in multiple comparisons (****p < 0.0001; **p < 0.01). In contrast, the CD-ZT3 group exhibited the lowest level of expression (mean ≈ 0.45 ± 0.08), representing a significantly reduced profile relative to groups with higher OCT4 activity (****p < 0.0001). No statistically significant differences were detected among CD-ZT15, TD-ZT3, and TD-ZT15 (ns), where OCT4 expression remained within a moderate range (≈ 0.70–0.85). Collectively, these findings indicate that temporal variation under CH conditions exerts a substantial regulatory influence on OCT4 transcript abundance, whereas analogous temporal shifts under CD or TD conditions do not elicit significant alterations in gene expression.

**Fig 2:**
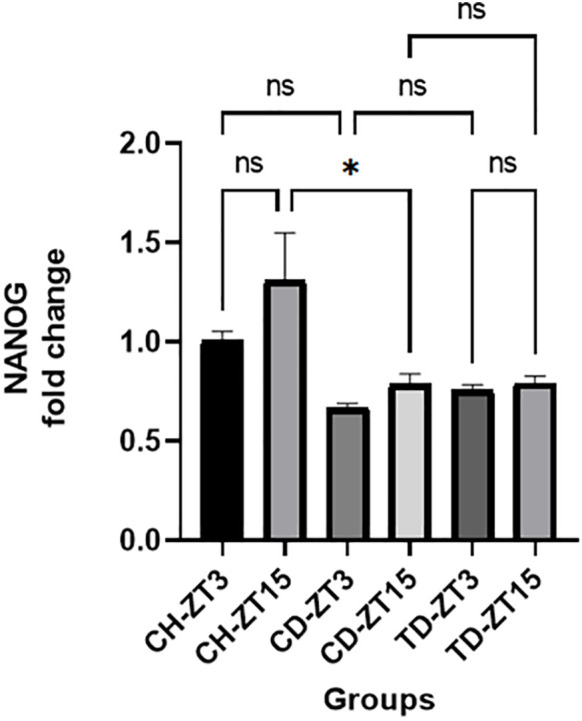
NANOG gene level expressed in different groups. Data are mean ± SD. pairwise comparisons Between bars indicates rate of significant difference at p\0.05.

Fig 2 illustrates the relative expression levels of the NANOG gene across the experimental groups. Analysis of NANOG expression revealed limited variability across most groups. The CH-ZT3 group demonstrated a mean expression of approximately 1.0 ± 0.10-fold, while the CH-ZT15 group showed a slight elevation to 1.3 ± 0.15-fold. The CD-ZT3 group exhibited reduced expression, approximately 0.65 ± 0.09-fold, which was significantly lower than CH-ZT15 (*p < 0.05). However, no significant differences were detected in comparisons involving any other groups (ns). The CD-ZT15, TD-ZT3, and TD-ZT15 groups displayed relatively stable expression profiles, with values ranging between 0.75–0.90-fold, indicating that NANOG expression was largely resistant to both conditional and temporal variation.

**Fig 3:**
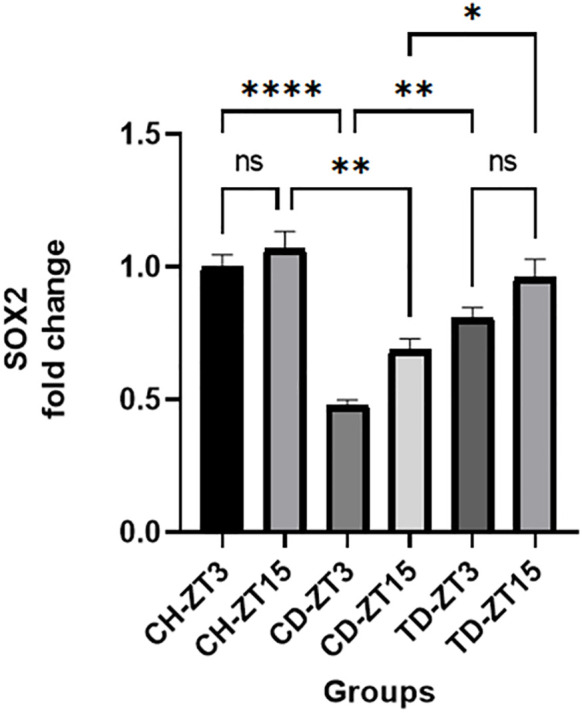
SOX2 gene level expressed in different groups. Data are mean ± SD. pairwise comparisons Between bars indicates rate of significant difference at p\0.05.

Fig 3 presents the relative expression levels of the SOX2 gene in the experimental groups Quantitative evaluation of SOX2 expression demonstrated substantial variation across the experimental conditions. The CH-ZT3 and CH-ZT15 groups exhibited comparable expression levels (≈ 1.0–1.1), with no statistically significant difference between them (ns). In contrast, the CD-ZT3 group showed a marked reduction in SOX2 expression (≈ 0.55 ± 0.07), and this decrease reached statistical significance relative to both CH groups (**p < 0.01; ****p < 0.0001). SOX2 levels increased in CD-ZT15 (≈ 0.85 ± 0.08), yielding a significant elevation compared with CD-ZT3 (**p < 0.01). Additionally, a modest but significant difference was observed between TD-ZT3 and TD-ZT15 (*p < 0.05), although both groups maintained expression values within a relatively narrow range (≈ 0.80–0.95). No significant difference was detected between CH-ZT15 and TD-ZT15 (ns). Overall, the data indicate that SOX2 expression is sensitive to condition-dependent modulation, with CD-ZT3 showing the most pronounced suppression, whereas temporal variability under CH and TD conditions produced only limited effects on transcriptional output.

**Fig 4:**
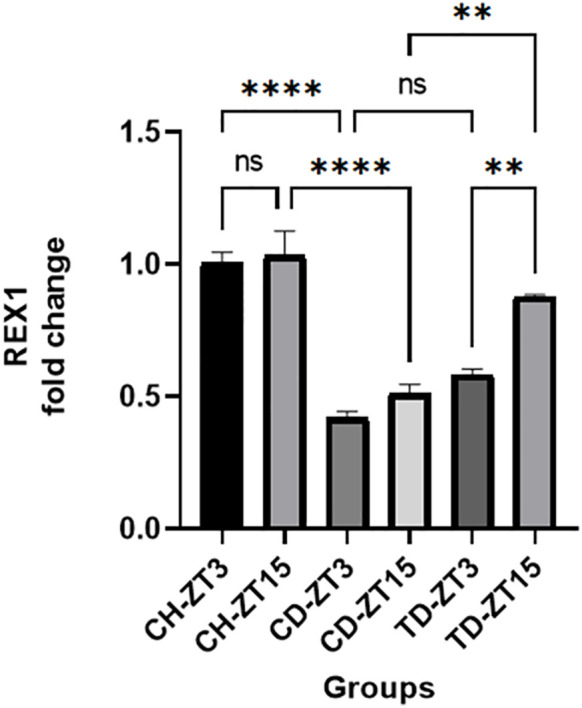
REX1 gene level expressed in different groups. Data are mean ± SD. pairwise comparisons between bars indicates rate of significant difference at p\0.05.

Fig 4 illustrates the relative expression levels of the REX1 gene across the experimental groups. Quantitative assessment of REX1 expression demonstrated substantial variability across the experimental groups. The CH-ZT3 and CH-ZT15 groups exhibited comparable REX1 levels (≈ 1.0–1.1), with no statistically significant difference between them (ns). In contrast, the CD-ZT3 group showed a markedly reduced expression level (≈ 0.50 ± 0.06), representing a significant downregulation relative to both CH groups (****p < 0.0001). A partial recovery of REX1 expression was observed in the CD-ZT15 group (≈ 0.75 ± 0.07), and this increase was statistically significant compared with CD-ZT3 (****p < 0.0001). The TD-ZT3 and TD-ZT15 groups exhibited intermediate expression values (≈ 0.85–1.05). The REX1 level in TD-ZT15 was significantly elevated relative to both CD-ZT3 and TD-ZT3 (**p < 0.01), while no significant difference was observed between CH-ZT15 and TD-ZT3 (ns). Overall, the data indicate that REX1 expression is strongly suppressed under CD-ZT3 conditions, whereas time-dependent variation under CD and TD conditions leads to partial or full restoration of transcriptional activity

## Discussion

The present findings demonstrate that diabetes is associated with a marked suppression of pluripotency-associated transcription factors, including OCT4, SOX2, and REX1, in bone tissue. These genes are essential regulators of cellular plasticity and regenerative capacity, and their down regulation suggests impaired stem cell like activity within the bone microenvironment under diabetic conditions. Previous studies have shown that hyperglycemia and oxidative stress disrupt stem cell maintenance pathways and reduce the expression of key pluripotency regulators, thereby limiting tissue repair potential [4,19].

Diabetes-induced skeletal deterioration is closely linked to imbalances in bone remodeling, characterized by reduced osteoblastic activity and enhanced osteoclastic resorption [3]. Chronic metabolic inflammation and impaired insulin signaling further contribute to decreased bone formation and reduced regenerative capacity [2]. In this context, the observed downregulation of pluripotency-associated genes may represent an additional molecular mechanism underlying compromised bone integrity in diabetes.

Importantly, our results indicate that aerobic exercise partially restores the expression of these genes, particularly when performed during the active circadian phase (ZT15). Exercise is known to activate multiple signaling pathways involved in cellular metabolism and regeneration, including AMPK, SIRT1, and Wnt/β-catenin pathways, which are critical for maintaining stem cell function and promoting tissue repair [20,21]. Furthermore, physical activity has been shown to act as a systemic zeitgeber, capable of re-synchronizing peripheral circadian clocks and improving metabolic homeostasis [22].

Circadian rhythm plays a fundamental role in regulating metabolic and transcriptional processes in bone tissue. Clock genes interact with osteogenic signaling pathways, influencing bone formation and resorption dynamics across the day-night cycle [23]. Disruption of circadian regulation, as observed in diabetic conditions, may therefore exacerbate skeletal fragility by impairing temporal coordination of regenerative pathways. The superior response observed in the ZT15 training group may reflect alignment between exercise-induced molecular signaling and endogenous circadian peak activity, thereby enhancing transcriptional responsiveness.

Overall, these findings support a model in which diabetes, circadian dysregulation, and physical activity converge to regulate bone tissue plasticity through pluripotency-associated gene networks. However, further studies integrating functional bone assessments are required to confirm whether these molecular changes translate into structural and biomechanical improvements.

The present study should be regarded as a pilot study because of the relatively small number of animals included in each experimental group. Therefore, although statistically significant molecular changes were observed, the findings should be interpreted as preliminary and require confirmation in larger animal studies with adequate statistical power.

### Limitation and future directions

A key limitation of the present study was the relatively small sample size, which limits the generalizability of the findings. Additionally, we did not evaluate histological changes, bone mineral density (BMD), or osteogenic differentiation markers, which limits the ability to directly infer improvements in bone regeneration or structural integrity. Therefore, while the observed changes in pluripotency-associated gene expression suggest potential molecular adaptations, their translational relevance to actual bone function remains indirect. Future studies should incorporate comprehensive structural and functional assessments, including bone histology, BMD analysis, and osteogenic markers such as osteoprotegerin (OPG) and osteopontin (OPN), to better elucidate the biological and clinical significance of these molecular findings.

## Conclusion

The present study demonstrates that diabetes is associated with a marked suppression of pluripotency-related gene expression in bone tissue, indicating a disruption of intrinsic regenerative molecular programs under metabolic stress. Aerobic exercise partially counteracts these alterations in a circadian-dependent manner, with greater efficacy observed during evening training (ZT15) compared to morning training (ZT3). These findings highlight the importance of temporal biology in modulating exercise-induced molecular responses and suggest that circadian alignment may be a critical determinant of metabolic adaptation in bone tissue.

## Supporting information

S1 File Raw data (ZIP archive containing Excel files) supporting the findings of this study. (RAR)
